# Supplementary material for: Plasma-to-tumour tissue integrated proteomics using nano-omics for biomarker discovery in glioblastoma
Source: Nat Commun. 2025 Apr 10;16:3412. doi: 10.1038/s41467-025-58252-0 (PMC11986092; doi:10.1038/s41467-025-58252-0)
Supplement: Supplementary file 1 — Supplementary Information [file 41467_2025_58252_MOESM1_ESM.pdf]

# Supplementary information

## Plasma-to-Tumour Tissue Integrated Proteomics Using Nano-omics for Biomarker Discovery in Glioblastoma

Xinming Liu[1,2,3], Hanan Abmanhal-Masarweh[1,3], Olivia Iwanowytsch[1,3], Emmanuel Okwelogu[1,3], Kiana Arashvand[2,3], Konstantina Karabatsou[4], Pietro Ivo D'Urso[4], Federico Roncaroli[5,6], Kostas Kostarelos[3,6,7,8,9], Thomas Kisby[2,3,6]*, Marilena Hadjidemetriou[1,3,6]*

[1] NanoOmics Lab, Division of Cancer Sciences, School of Medical Sciences, Faculty of Biology, Medicine and Health, The University of Manchester, Manchester, UK

[2] NanoTherapeutics Lab, Division of Cell Matrix Biology and Regenerative Medicine, School of Biological Sciences, Faculty of Biology, Medicine and Health, University of Manchester, Manchester, UK

[3] Centre for Nanotechnology in Medicine, Faculty of Biology, Medicine and Health, The University of Manchester, Manchester, UK

[4] Department of Neurosurgery, Manchester Centre for Clinical Neurosciences, Salford Royal NHS Foundation Trust, Manchester, UK

[5] Division of Neuroscience, School of Biological Sciences, Faculty of Biology, Medicine and Health, The University of Manchester, Manchester, UK

[6] Geoffrey Jefferson Brain Research Centre, Manchester Academic Health Science Centre, Northern Care Alliance NHS Foundation Trust, The University of Manchester, Manchester, UK

[7] Nanomedicine Lab, Catalan Institute of Nanoscience and Nanotechnology (ICN2), CSIC and BIST, Campus UAB, 08193 Barcelona, Spain

[8] Institute of Neuroscience, Universitat Autònoma de Barcelona, 08913 Barcelona, Spain

[9] Institució Catalana de Recerca i Estudis Avançats (ICREA), Pg. Lluís Companys 23, Barcelona, Spain

* Email: thomas.kisby@manchester.ac.uk; marilena.hadjidemetriou@manchester.ac.uk

# Supplementary figure 1

**a**

| Lipid composition | Lipid molar ratio | Mean hydrodynamic diameter (nm) | Z-potential (mV) | Polydispersity Index (PDI) |
|---|---|---|---|---|
| HSPC:Chol:DSPE-PEG2000 | 56.3:38.2:5.5 | 110.3 ± 1.682 | -34.47 ± 0.7833 | 0.041 ± 0.011 |

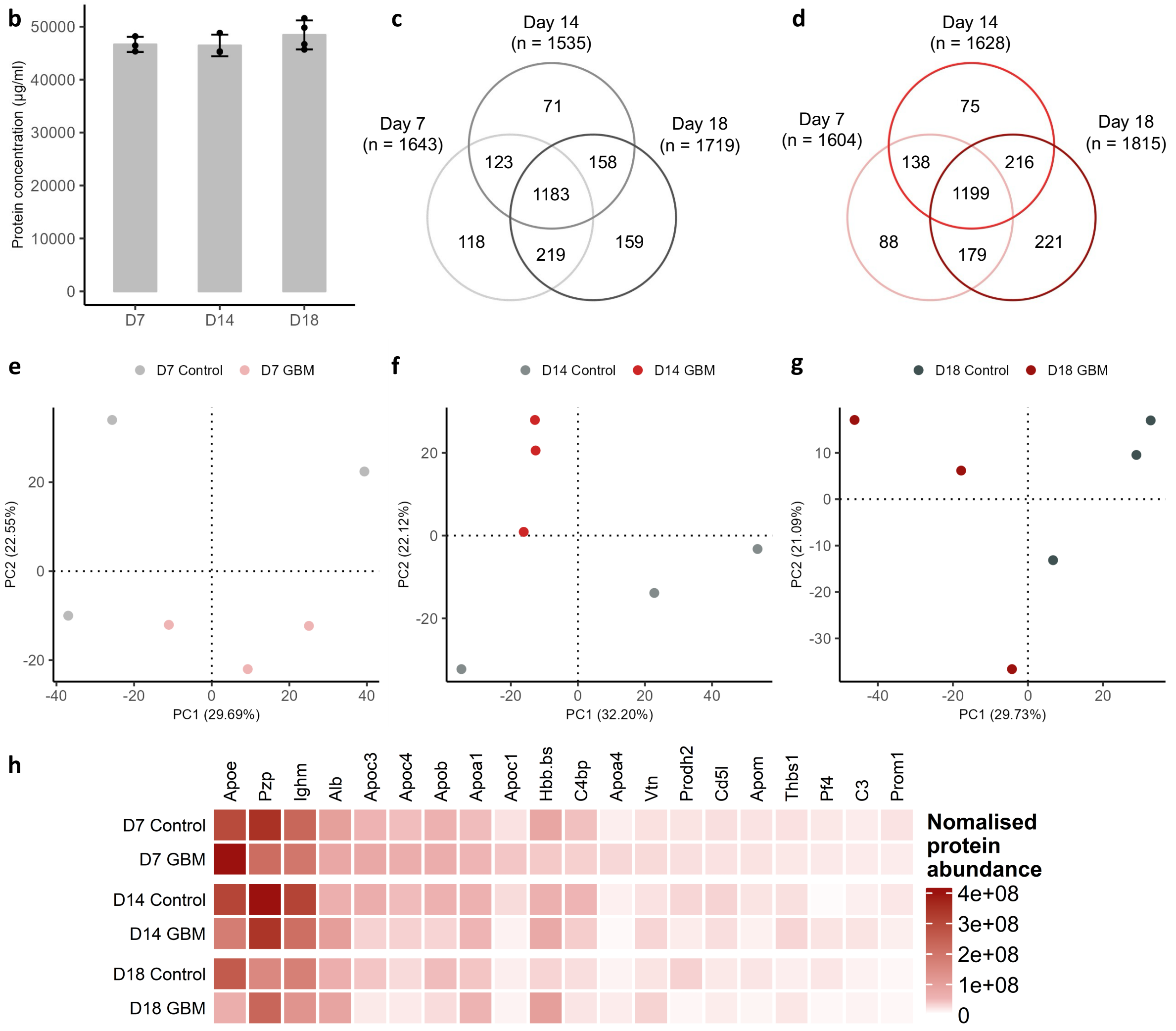


**Fig.S1 I Nanotechnology-enabled plasma proteomics in the GL261 mouse model of GB.**
**a,** Physicochemical characterisation of liposome NPs employed in this study. **b,** Bar chart displays the total protein concentration in plasma samples obtained from tumour-bearing mice at D7, D14 and D18 post-tumour inoculation. Error bars represent the mean +/- SD of n=3 biological replicates for D7 and D14, n=4 biological replicates for D18. **c, d,** Venn diagrams showing the total number of corona proteins identified by LC-MS/MS at the three time points (D7, D14, and D18) of investigation in sham-injected control and GB-bearing mice, respectively. **e, f, g,** PCA score plots of proteomics data showing distinct grouping of corona samples in control and GB-bearing mice (grey and red dots, respectively) at D7, D14, and D18. **h,** Top 20 most abundant proteins identified by LC-MS/MS analysis of corona samples recovered from sham-injected control and GB-bearing mice at D7, D14, and D18. Source data for Fig.S1b,h are provided as a Source Data file.

# Supplementary figure 2

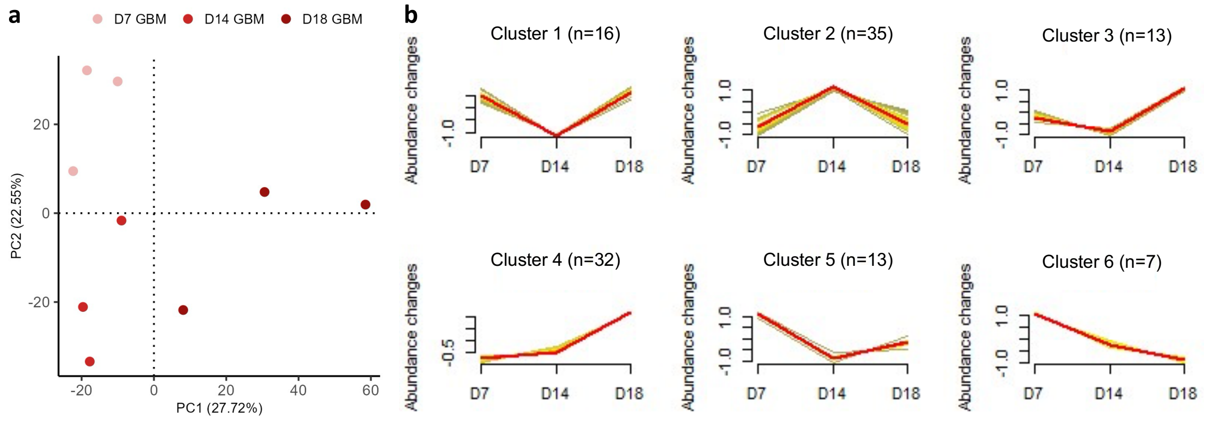


**c**

List of disease monitoring proteins

Upregulated (n = 32)

| Gene name | Description | D7 vs D14 Fold change | D7 vs D14 P-value | D14 vs D18 Fold change | D14 vs D18 P-value |
|---|---|---|---|---|---|
| Agpat2 | 1-acyl-sn-glycerol-3-phosphate acyltransferase beta | 1.368 | 0.043 | 2.586 | 0.049 |
| Amy2 | Pancreatic alpha-amylase | 4.916 | 0.008 | 2.990 | 0.020 |
| Ass1 | Argininosuccinate synthase | 1.946 | 0.044 | 3.536 | 0.008 |
| Canx | Calnexin | 1.332 | 0.013 | 3.086 | 0.042 |
| Cel | Bile salt-activated lipase | 2.148 | 0.012 | 3.892 | 0.003 |
| Cela1 | Chymotrypsin-like elastase family member 1 | 3.709 | 0.027 | 3.602 | 0.031 |
| Cpb1 | Carboxypeptidase B1 (Tissue), isoform CRA_b | 649.543 | 0.001 | 13.515 | 0.005 |
| Dmbt1 | Deleted in malignant brain tumours 1 protein | 6.496 | 0.023 | 2.725 | 0.046 |
| Elovl2 | Elongation of very long chain fatty acids protein 2 | 1.778 | 0.007 | 3.887 | 0.028 |
| Erlin2 | Erlin-2 | 2.716 | 0.031 | 5.446 | 0.015 |
| Gm10320 | Protein transport protein Sec61 subunit beta | 1.404 | 0.035 | 3.307 | 0.003 |
| Hspd1 | 60 kDa heat shock protein, mitochondrial | 3.259 | 0.006 | 7.124 | 0.002 |
| Krt8 | Keratin, type II cytoskeletal 8 | 2.813 | 0.005 | 4.950 | 0.008 |
| Lmf1 | Lipase maturation factor 1 | 2.683 | 0.031 | 7.643 | 0.013 |
| Lmf2 | Lipase maturation factor 2 | 2.336 | 0.015 | 5.085 | 0.004 |
| Mlec | Malectin | 1.820 | 0.032 | 5.633 | 0.012 |
| Nat8f3 | N-acetyltransferase 8 (GCN5-related) family member 3 | Infinity | < 0.001 | 18.429 | 0.001 |
| Os9 | Protein OS-9 | Infinity | 0.001 | 40.047 | 0.012 |
| Rpn1 | Dolichyl-diphosphooligosaccharide--protein glycosyltransferase subunit 1 | 2.233 | 0.008 | 6.354 | 0.010 |
| Rpn2 | Dolichyl-diphosphooligosaccharide--protein glycosyltransferase subunit 2 | 1.949 | 0.011 | 4.969 | 0.014 |
| Sec61a1 | SEC61 translocon subunit alpha 1 | 1.670 | 0.026 | 4.519 | 0.011 |
| Sec63 | Translocation protein SEC63 homolog | 2.124 | 0.035 | 8.658 | 0.016 |
| Soat2 | Sterol O-acyltransferase 2 | 3.892 | 0.028 | 12.174 | 0.005 |
| Sod1 | Superoxide dismutase [Cu-Zn] | 1.188 | 0.042 | 1.330 | 0.045 |
| Stt3a | STT3 oligosaccharyltransferase complex catalytic subunit A | 2.365 | 0.032 | 5.379 | 0.022 |
| Synj2bp | Synaptojanin-2-binding protein | 1.859 | 0.009 | 3.407 | 0.023 |
| Tap2 | Transporter 2 ATP-binding cassette sub-family B | 1.856 | 0.037 | 8.241 | 0.004 |
| Tm6sf2 | Transmembrane 6 superfamily member 2 | 2.788 | 0.049 | 4.951 | 0.018 |
| Tmc6 | Transmembrane channel-like protein | 2.048 | 0.014 | 2.172 | 0.017 |
| Ugt2b1 | UDP-glucuronosyltransferase | 1.822 | 0.007 | 3.088 | 0.035 |
| Vwa8 | von Willebrand factor A domain-containing protein 8 | 1.243 | 0.023 | 2.130 | 0.008 |
|  | Alpha-amylase | 4.916 | 0.008 | 2.990 | 0.020 |

Downregulated (n = 7)

| Gene name | Description | D7 vs D14 Fold change | D7 vs D14 P-value | D14 vs D18 Fold change | D14 vs D18 P-value |
|---|---|---|---|---|---|
| Ahsg | Alpha-2-HS-glycoprotein | 1.331 | 0.025 | 1.344 | 0.031 |
| Cacna2d1 | Voltage-dependent calcium channel subunit alpha-2/delta-1 | 1.961 | 0.003 | 1.308 | 0.026 |
| Col1a1 | Collagen alpha-1(I) chain | 1.732 | 0.048 | 2.009 | 0.002 |
| Colec11 | Collectin-11 | 1.642 | 0.002 | 2.077 | 0.004 |
| Plin3 | Perilipin-3 | 2.737 | 0.018 | 4.216 | 0.014 |
| Rras2 | Ras-related protein R-Ras2 | 1.470 | 0.002 | 1.278 | 0.018 |
| Tgfb1 | Transforming growth factor beta-1 | 1.552 | 0.028 | 1.300 | 0.035 |

**Fig.S2 | Longitudinal monitoring of the GB-specific plasma proteome in GL261-bearing mice.**
**a**, PCA score plots of proteomic data showing distinct grouping of corona samples at D7, D14, and D18. **b**, Fuzzy c-means clustering of all n=116 disease monitoring proteins throughout the three time points was performed based on normalised protein abundance, resulting in 6 different trends (clusters). Each line indicates the relative abundance of each protein. Proteins with low deviation were indicated by warm coloured traces (red/orange), while proteins with high deviation were coloured in cold colours (grey/beige). Full lists of all n=116 disease monitoring proteins are shown in Supplementary Data 6. **c**, List of n=32 upregulated (cluster 4) and n=7 downregulated (cluster 6) disease monitoring proteins. P-values are calculated by one-way ANOVA.

# Supplementary figure 3

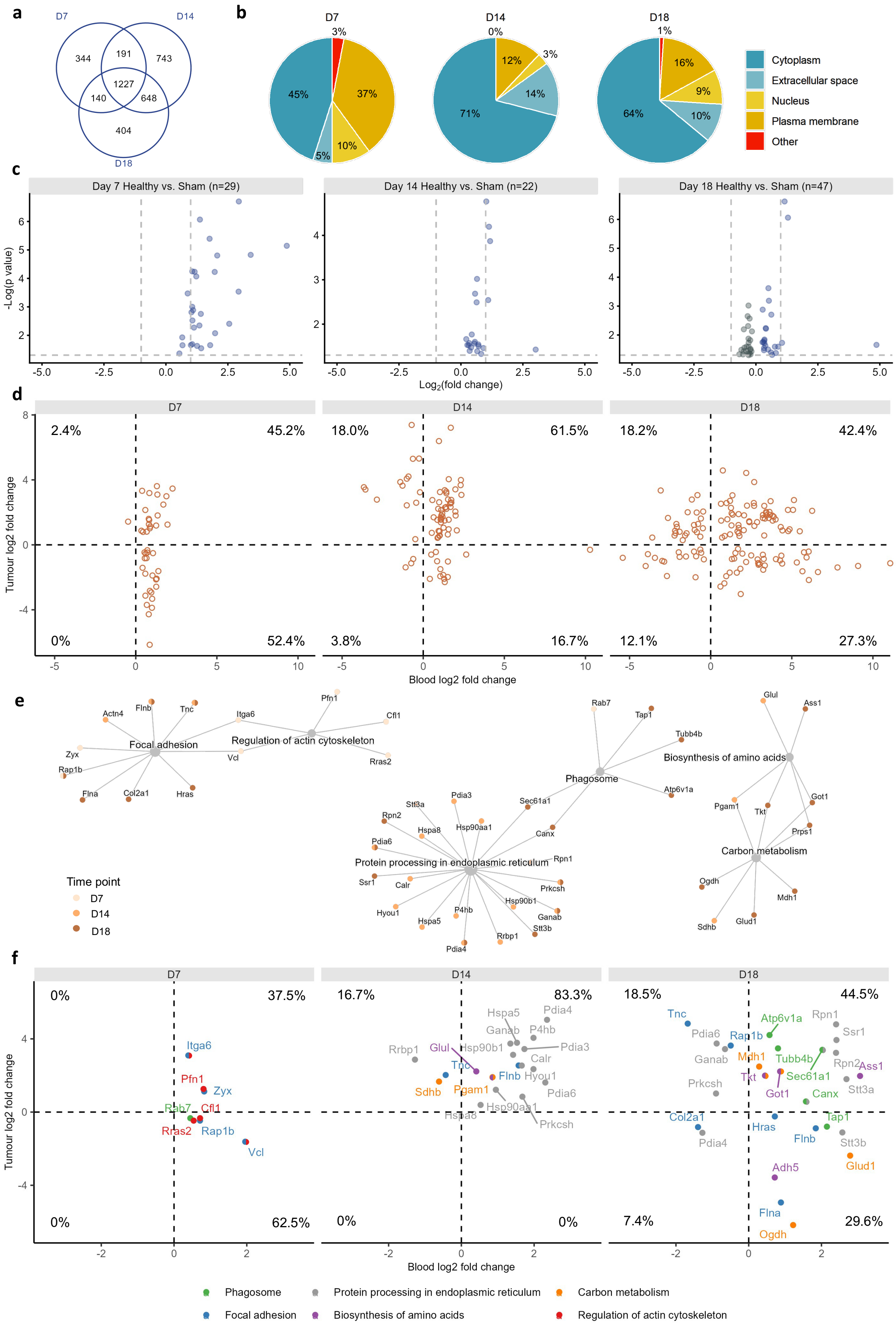

**Fig.S3 | Integrative analysis of the plasma and tumour tissue proteomes in GL261-bearing mice.**
**a,** Venn diagram illustrates the number of common and unique differentially abundant proteins (DAPs with a one-way ANOVA p-value < 0.05) between healthy and tumour tissues identified at D7, D14 and D18. **b,** Ingenuity Pathway Analysis (IPA) of the overlapping DAPs, identified in both plasma and tumour tissue at D7, D14 and D18. Pie charts illustrate protein classification by cellular location. **c,** Volcano plots showing the relationship between fold change and significance for DAPs identified through comparisons between sham-injected hemisphere and contralateral (non-injected) hemisphere from sham-injected control mice for the three different time points. DAPs with a one-way ANOVA p-value<0.05 are shown. Full lists of proteins are shown in Supplementary Data 7-9. **d,** Scatter plots display the relationship between the fold change in plasma and tumour for the commonly identified DAPs at D7, D14 and D18 post-tumour inoculation. **e,** Gene-concept network of common plasma-to-tumour tissue DAPs involved in six key KEGG pathways. Among these pathways, five were identified in at least two of the three time points investigated. Additionally, the regulation of actin cytoskeleton, included due to its potential as an early detection biomarker source, is represented in the network. Concept nodes, represent the enriched pathways and are sized according to the protein count involved in that pathway, linking to the protein nodes involved in each pathway. Colour of the protein nodes indicate the three time points. **f,** Scatter plots display the relationship between the fold change in plasma and tumour for the commonly identified DAPs that were involved in the six KEGG pathways. Source data for Fig.S3b are provided as a Source Data file.

# Supplementary figure 4

a

| Patient ID | Diagnosis | Sex | Age range | Tumour site | Notes |
|---|---|---|---|---|---|
| Patient 1 | Glioblastoma (WHO grade IV) | Male | 50-60 | Right parietal lobe | IDH1/2 wide-type |
| Patient 2 | Glioblastoma (WHO grade IV) | Male | 60-70 | Right parietal and right temporal lobe | IDH1/2 wide-type |
| Patient 3 | Glioblastoma (WHO grade IV) | Female | 60-70 | Left frontal lobe | IDH1/2 wide-type |
| Patient 4 | Glioblastoma (WHO grade IV) | Male | 70-80 | Right parietal lobe | IDH1/2 wide-type |
| Patient 5 | Recurrent glioblastoma (WHO grade IV) | Female | 60-70 | Left parietal lobe | IDH1/2 wide-type |
| Patient 6 | Glioblastoma (WHO grade IV) | Female | 50-60 | Left thalamus | IDH1/2 wide-type |
| Patient 7 | Recurrent glioblastoma (WHO grade IV)* | Male | 30-40 | Left parietal lobe | IDH1(R132H) mutant |
| Patient 8 | Glioblastoma (WHO grade IV) | Female | 50-60 | Right temporal lobe | IDH1/2 wide-type |
| Patient 9 | Glioblastoma (WHO grade IV) | Male | 50-60 | Right parietal lobe | IDH1/2 wide-type |
| Patient 10 | Glioblastoma (WHO grade IV) | Male | 60-70 | Right parietal lobe | IDH1/2 wide-type |

b

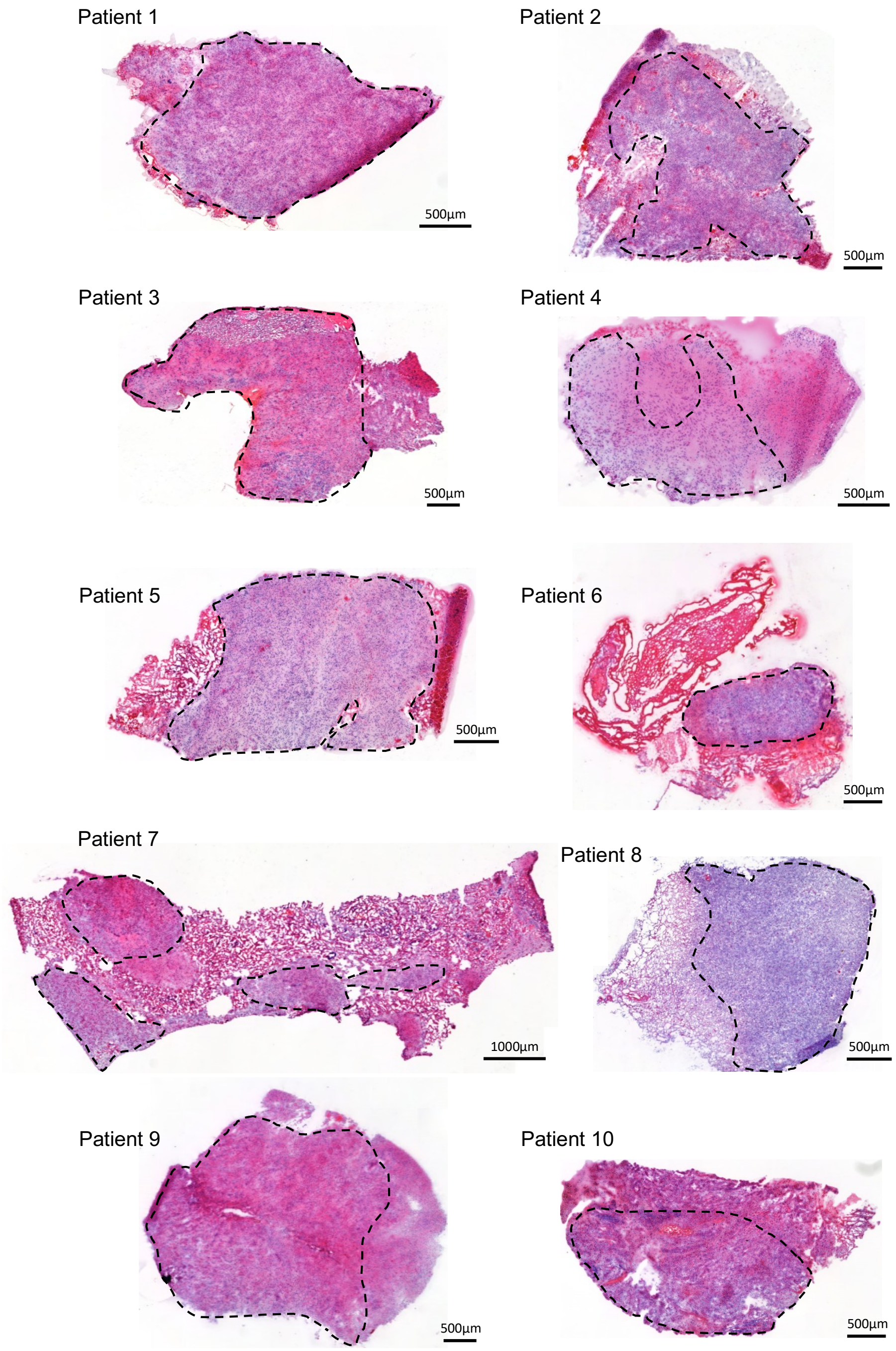


**Fig.S4 | Human clinical validation of the plasma-to-tumour tissue integrative proteomic analysis pipeline.** **a,** Table with patient cohort clinical information. The cases have been classified according to the 5th edition of the WHO Classification of Central Nervous System Tumours (2021). * The tissue sample was collected at the time of the second operation when the tumour progressed to grade 4. **b,** H&E-stained snap-frozen human GB tissue samples. Dashed line indicates tumour area. Equal total area (10 $mm^2$) of tumour tissue was collected by laser microdissection for each patient prior to LC:MS/MS analysis.

# Supplementary figure 5

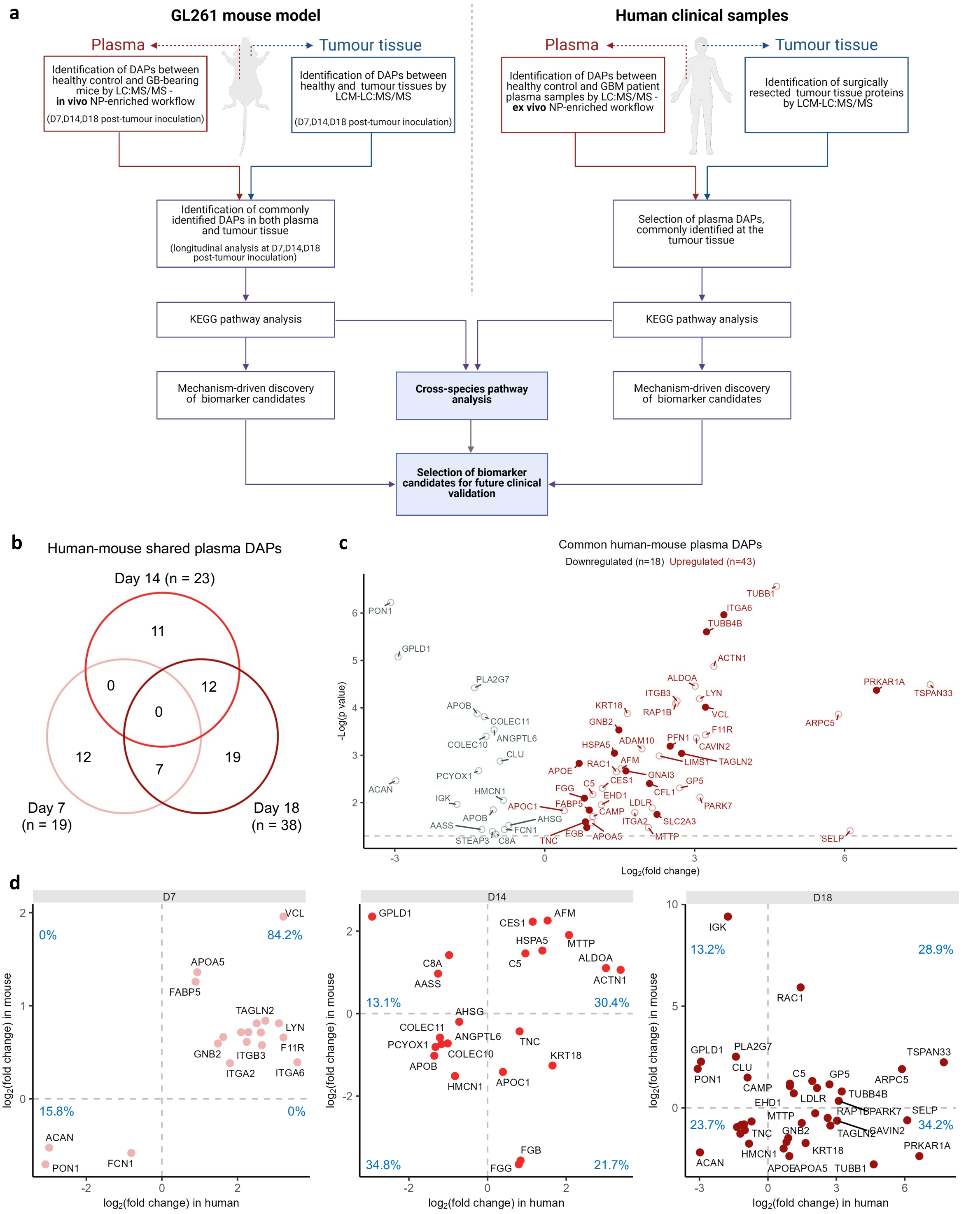


**Fig.S5 | Human-mouse cross-species validation of the proteomics data. a,** The flow chart summarizes the integration of proteomics data obtained by the analysis of plasma and GB tumour tissue proteomes in the GL261 mouse model and human patients aiming to elucidate potential biomarker candidates via a mechanism-driven biomarker discovery approach. Created in BioRender. Hadjidemetriou, M. (2025) https://BioRender.com/u99u306. **b,** Venn diagram reports the number of unique and shared plasma DAPs between human and mouse species at D7, D14, D18. **c**, Volcano plot shows the p-value (one-way ANOVA) and fold change of n=61 human plasma-to-tumour tissue shared proteins that were also identified in mouse plasma, filled dots (n=16) indicate proteins that were also identified as DAPs in the mouse tumour tissue. **d,** Scatter plots displaying the relationship between the fold changes of commonly identified DAPs in mice (at D7, D14 and D18) and humans.

# Supplementary figure 6

| Gene name | Description | Plasma *p* value | Plasma fold change | Previously reported in GBM plasma | Previously reported in plasma of other cancers |
|---|---|---|---|---|---|
| **Human plasma-to-tumour tissue shared proteins** | | | | | |
| **ACTB** | Actin, cytoplasmic 1 | 3.67E-05 | 4.31 | No | Yes |
| **ACTR3** | ARP3 actin-related protein 3 homolog | 6.80E-05 | 6.36 | No | No |
| **ARPC1B** | Actin-related protein 2/3 complex subunit 1B | 2.80E-05 | 11.85 | No | No |
| **ARPC2** | Actin-related protein 2/3 complex subunit 2 | 1.65E-05 | 8.76 | No | Yes |
| **ARPC4** | Actin-related protein 2/3 complex subunit 4 | 1.85E-04 | 30.36 | No | No |
| **CDC42** | Cell division control protein 42 homolog | 3.07E-04 | 2.55 | No | Yes |
| **COL6A2** | Collagen alpha-2(VI) chain | 1.22E-02 | -1.53 | No | No |
| **CORO1A** | Coronin | 2.37E-05 | 8.74 | No | Yes |
| **CYFIP1** | Cytoplasmic FMR1-interacting protein 1 | 2.37E-05 | 7.93 | No | No |
| **ENO1** | Enolase 1 (Alpha) | 1.23E-04 | 3.59 | No | Yes |
| **F2** | Prothrombin | 4.66E-03 | 3.27 | Yes | Yes |
| **FCER1G** | High affinity immunoglobulin epsilon receptor subunit gamma | 3.97E-05 | 3.26 | No | No |
| **FGA** | Fibrinogen alpha chain | 2.95E-02 | 1.88 | No | Yes |
| **FN1** | Fibronectin 1 | 1.15E-03 | -2.26 | No | Yes |
| **GAPDH** | Glyceraldehyde-3-phosphate dehydrogenase | 1.24E-04 | 3.6 | No | Yes |
| **GNAI2** | Guanine nucleotide-binding protein G(i) subunit alpha-2 | 2.20E-02 | 1.71 | No | Yes |
| **ILK** | Integrin-linked protein kinase | 2.70E-06 | 7.79 | No | Yes |
| **ITGB1** | Integrin beta-1 | 6.27E-04 | 3.03 | Yes | Yes |
| **MYH9** | Myosin heavy polypeptide 9 | 1.97E-05 | 4.19 | Yes | Yes |
| **MYL12A** | Myosin regulatory light chain 12A | 1.17E-03 | 12.78 | No | No |
| **NCKAP1** | Nck-associated protein 1 | 1.22E-02 | 11.91 | No | No |
| **PFKL** | ATP-dependent 6-phosphofructokinase | 8.44E-03 | Infinity | No | No |
| **PGK1** | Phosphoglycerate kinase 1 | 1.31E-05 | 8.24 | Yes | Yes |
| **RAC2** | Ras-related C3 botulinum toxin substrate 2 | 3.46E-05 | 3.4 | No | No |
| **TLN1** | Talin-1 | 3.10E-06 | 14.98 | No | Yes |
| **TPI1** | Triosephosphate isomerase | 1.25E-02 | 3.62 | Yes | Yes |
| **TUBA4A** | Tubulin alpha-4A chain | 8.06E-07 | 35.37 | No | Yes |
| **TUBB** | Tubulin beta chain | 3.80E-05 | 5.32 | No | Yes |
| **VASP** | Vasodilator-stimulated phosphoprotein isoform 1 | 1.04E-03 | 6.99 | No | Yes |
| **VWF** | von Willebrand factor | 2.90E-07 | 29.94 | Yes | Yes |
| **WASF2** | Wiskott-Aldrich syndrome protein family member 2 | 1.45E-05 | 7.57 | No | No |
| **Human plasma-to-tumour tissue shared proteins (identified in mouse plasma)** | | | | | |
| **Gene name** | **Description** | **Plasma *p* value** | **Plasma fold change** | **Previously reported in GBM plasma** | **Previously reported in plasma of other cancers** |
| **ALDOA** | Fructose-bisphosphate aldolase A | 3.49E-05 | 8.06 | Yes | Yes |
| **ARPC5** | Actin-related protein 2/3 complex subunit 5 | 1.36E-04 | 58.68 | No | No |
| **C5** | Complement C5 | 6.76E-03 | 1.95 | Yes | Yes |
| **C8A** | Complement component C8 alpha chain | 4.62E-02 | -1.97 | No | Yes |
| **ITGA2** | Integrin alpha-2 | 1.61E-02 | 3.48 | No | Yes |
| **LYN** | Tyrosine-protein kinase Lyn | 6.47E-05 | 8.54 | No | No |
| **RAC1** | Ras-related C3 botulinum toxin substrate 1 | 2.23E-03 | 2.7 | No | Yes |
| **Human plasma-to-tumour tissue shared proteins (identified in mouse plasma and tumour tissue)** | | | | | |
| **Gene name** | **Description** | **Plasma *p* value** | **Plasma fold change** | **Previously reported in GBM plasma** | **Previously reported in plasma of other cancers** |
| **CFL1** | Cofilin-1 | 3.94E-03 | 4.27 | No | No |
| **FGB** | Fibrinogen beta chain | 3.34E-02 | 1.79 | No | Yes |
| **FGG** | Fibrinogen gamma chain | 8.02E-03 | 1.73 | No | Yes |
| **GNAI3** | Guanine nucleotide-binding protein G(k) subunit alpha | 2.14E-03 | 3.08 | No | No |
| **ITGA6** | Integrin alpha-6 | 1.09E-06 | 11.98 | No | Yes |
| **PFN1** | Profilin-1 | 6.42E-04 | 5.69 | No | Yes |
| **RAP1B** | RAP1B, member of RAS oncogene family | 8.18E-05 | 6.14 | Yes | Yes |
| **TNC** | Tenascin C | 2.54E-02 | 1.76 | Yes | Yes |
| **TUBB4B** | Tubulin beta-4B chain | 2.49E-06 | 9.42 | No | No |
| **VCL** | Vinculin | 9.67E-05 | 9.29 | No | Yes |

**Fig.S6 I Integrative analysis of the plasma and tumour tissue proteomes in patients with high grade gliomas.** Full list of the n=48 human plasma-to-tumour tissue shared proteins involved in 7 pathways found to be shared between both human and mouse species: the regulation of actin cytoskeleton, focal adhesion, platelet activation, leukocyte transendothelial migration, biosynthesis of amino acids, carbon metabolism and phagosome. P-values are calculated by one-way ANOVA. Out of the 48 human DAPs mapped to the seven pathways, 17 were also found to exhibit differential abundance in the plasma of GL261 tumour-bearing mice, with

10 of these also identified in the mouse tumour tissue. Proteins not previously reported at the plasma level in GB, or any other cancer type are highlighted in blue.
