# Supplementary material for: Plasma-to-tumour tissue integrated proteomics using nano-omics for biomarker discovery in glioblastoma
Source: Nat Commun. 2025 Apr 10;16:3412. doi: 10.1038/s41467-025-58252-0 (PMC11986092; doi:10.1038/s41467-025-58252-0)
Supplement: Supplementary file 2 — Description of Additional Supplementary Files [file 41467_2025_58252_MOESM2_ESM.pdf]

Description of Additional Supplementary Files

Supplementary Data 1. Full list of blood proteins identified by Progenesis analysis to be differentially abundant at D7 time point between GBM-bearing mice and control mice. Only proteins with a p value < 0.05 by one-way ANOVA test are shown.

Supplementary Data 2. Full list of blood proteins identified by Progenesis analysis to be differentially abundant at D14 time point between GBM-bearing mice and control mice. Only proteins with a p value < 0.05 by one-way ANOVA test are shown.

Supplementary Data 3. Full list of blood proteins identified by Progenesis analysis to be differentially abundant at D18 time point between GBM-bearing mice and control mice. Only proteins with a p value < 0.05 by one-way ANOVA test are shown.

Supplementary Data 4. Full list of blood proteins identified by Progenesis analysis to be differentially abundant between D7 and D14 time point in GBM-bearing mice. Only proteins with a p value < 0.05 by one-way ANOVA test are shown.

Supplementary Data 5. Full list of blood proteins identified by Progenesis analysis to be differentially abundant between D14 and D18 time point in GBM-bearing mice. Only proteins with a p value < 0.05 by one-way ANOVA test are shown.

Supplementary Data 6. Full list of disease monitoring proteins identified by Progenesis analysis to be differentially abundant between D7 and D14 time point, as well as between D14 and D18 time point in GBM-bearing mice. Only proteins with a p value < 0.05 by one-way ANOVA test are shown.

Supplementary Data 7. Full list of proteins identified by Progenesis analysis to be differentially abundant at D7 time point between sham injection site and healthy brain. Only proteins with a p value < 0.05 by one-way ANOVA test are shown.

Supplementary Data 8. Full list of proteins identified by Progenesis analysis to be differentially abundant at D14 time point between sham injection site and healthy brain. Only proteins with a p value < 0.05 by one-way ANOVA test are shown.

Supplementary Data 9. Full list of proteins identified by Progenesis analysis to be differentially abundant at D18 time point between sham injection site and healthy brain. Only proteins with a p value < 0.05 by one-way ANOVA test are shown.

Supplementary Data 10. Full list of proteins identified by Progenesis analysis to be differentially abundant at D7 time point between tumour tissue and healthy brain. Only proteins with a p value < 0.05 by one-way ANOVA test are shown. Overlapping DAPs also identified by the proteomics analysis of plasma samples are highlighted in orange.

Supplementary Data 11. Full list of proteins identified by Progenesis analysis to be differentially abundant at D14 time point between tumour tissue and healthy brain. Only proteins with a p value < 0.05 by one-way ANOVA test are shown. Overlapping DAPs also identified by the proteomics analysis of plasma samples are highlighted in orange.

Supplementary Data 12. Full list of proteins identified by Progenesis analysis to be differentially abundant at D18 time point between tumour tissue and healthy brain. Only proteins with a p value < 0.05 by one-way ANOVA test are shown. Overlapping DAPs also identified by the proteomics analysis of plasma samples are highlighted in orange.

Supplementary Data 13. Full list of commonly identified differential abundant proteins identified by Progenesis analysis between mouse blood and brain tissue. Only proteins with a p value < 0.05 by one-way ANOVA test are shown.

Supplementary Data 14. Full list of blood proteins identified by Progenesis analysis to be differentially abundant between n = 10 GBM patients and n = 10 healthy controls. Only proteins with a q value < 0.05 by one-way ANOVA test are shown.

Supplementary Data 15. Full list of tissue proteins identified in GBM tumour biopsies from n = 10 GBM patients.

Supplementary Data 16. Full list of blood differentially abundant proteins between GBM patients and healthy controls that have also been identified in the GBM tumour proteome. P values calculated by one-way ANOVA tests.
