# Supplementary material for: Plasma-to-tumour tissue integrated proteomics using nano-omics for biomarker discovery in glioblastoma
Source: Nat Commun. 2025 Apr 10;16:3412. doi: 10.1038/s41467-025-58252-0 (PMC11986092; doi:10.1038/s41467-025-58252-0)
Supplement: Supplementary file 19 — Reporting Summary [file 41467_2025_58252_MOESM19_ESM.pdf]

nature portfolio

Corresponding author(s): Marilena Hadjidemetriou

Last updated by author(s): Feb 18, 2025

Reporting Summary

Nature Portfolio wishes to improve the reproducibility of the work that we publish. This form provides structure for consistency and transparency in reporting. For further information on Nature Portfolio policies, see our Editorial Policies and the Editorial Policy Checklist.

Statistics

For all statistical analyses, confirm that the following items are present in the figure legend, table legend, main text, or Methods section.

n/a	Confirmed	
[ ]	[x]	The exact sample size (*n*) for each experimental group/condition, given as a discrete number and unit of measurement
[ ]	[x]	A statement on whether measurements were taken from distinct samples or whether the same sample was measured repeatedly
[ ]	[x]	The statistical test(s) used AND whether they are one- or two-sided<br>*Only common tests should be described solely by name; describe more complex techniques in the Methods section.*
[x]	[ ]	A description of all covariates tested
[ ]	[x]	A description of any assumptions or corrections, such as tests of normality and adjustment for multiple comparisons
[ ]	[x]	A full description of the statistical parameters including central tendency (e.g. means) or other basic estimates (e.g. regression coefficient) AND variation (e.g. standard deviation) or associated estimates of uncertainty (e.g. confidence intervals)
[ ]	[x]	For null hypothesis testing, the test statistic (e.g. *F*, *t*, *r*) with confidence intervals, effect sizes, degrees of freedom and *P* value noted<br>*Give P values as exact values whenever suitable.*
[x]	[ ]	For Bayesian analysis, information on the choice of priors and Markov chain Monte Carlo settings
[x]	[ ]	For hierarchical and complex designs, identification of the appropriate level for tests and full reporting of outcomes
[x]	[ ]	Estimates of effect sizes (e.g. Cohen's *d*, Pearson's *r*), indicating how they were calculated

Our web collection on statistics for biologists contains articles on many of the points above.

Software and code

Policy information about availability of computer code

Data collection	Raw mass data were collected using Progenesis QI for proteomics software (v. 3.0; Nonlinear Dynamics).
Data analysis	Raw mass data were processed using Progenesis QI for proteomics software (v. 3.0; Nonlinear Dynamics). The "clusterProfiler" package (v4.10.0) from Bioconductor was used to perform overrepresentation analysis. All statistical analyses and data plotting were performed using R (v. 4.2.2), RStudio (v. 2023.06.0, Posit Software) and GraphPad Prism 9 software package (GraphPad Software).

For manuscripts utilizing custom algorithms or software that are central to the research but not yet described in published literature, software must be made available to editors and reviewers. We strongly encourage code deposition in a community repository (e.g. GitHub). See the Nature Portfolio guidelines for submitting code & software for further information.

Data

Policy information about availability of data

All manuscripts must include a data availability statement. This statement should provide the following information, where applicable:

- Accession codes, unique identifiers, or web links for publicly available datasets
- A description of any restrictions on data availability
- For clinical datasets or third party data, please ensure that the statement adheres to our policy

The data that support the findings of this study are available in the article and supplementary information files. The mass spectrometry proteomics data generated in this study have been deposited to the ProteomeXchange Consortium via the PRIDE partner repository with the accession code PXD060393. Source data is available for Figures 1c, 2b-e, 3a-e, 4b-c and 5b-c and Supplementary Figures 1b-h, 2a-b, 3a-d, 3f and 5b-d in the associated source data file and Supplementary

Table file.

Research involving human participants, their data, or biological material

Policy information about studies with human participants or human data. See also policy information about sex, gender (identity/presentation), and sexual orientation and race, ethnicity and racism.

Reporting on sex and gender	Matched plasma and tumour tissue samples were obtained from human high-grade gliomas patients (n=6 male and n=4 female). Sex- and age-matched healthy controls were used . We acknowledge that the small sample size in our human cohort experiment (n=6 males; n=4 females) limits our ability to draw definitive conclusions regarding the sex-specificity of the candidate biomarkers identified.
Reporting on race, ethnicity, or other socially relevant groupings	Ethnicity was not considered as a variable in the analysis of the samples. This decision was made to focus the development of plasma to tumour tissue integrative proteomics pipeline. Given the limited access to paired plasma-to-tumour tissue samples, conclusions drawn from this research may not account for potential variations attributable to ethnic differences.
Population characteristics	Matched plasma and tumour tissue samples obtained from 10 patients with high grade gliomas were analysed.The cases have been classified according to the 5th edition of the WHO Classification of Central Nervous System Tumours (2021). This information is provided in (Supplementary Figure 4a).
Recruitment	Participants were not specifically recruited for this study. Frozen plasma aliquots and tumour tissue from n=10 GB patients were provided by Manchester Centre of Clinical Neuroscience.
Ethics oversight	Frozen plasma aliquots and tumour tissue from n=10 GB patients (n=9 primary and n=1 recurrent) were provided by Manchester Centre of Clinical Neuroscience following project review and ethical approval from at the Northern Care Alliance Research Collection (IRAS: 145069 – REC reference 19NCABB001_001). Informed consent was obtained from all participants.

Note that full information on the approval of the study protocol must also be provided in the manuscript.

Field-specific reporting

Please select the one below that is the best fit for your research. If you are not sure, read the appropriate sections before making your selection.

- [x] Life sciences
- [ ] Behavioural & social sciences
- [ ] Ecological, evolutionary & environmental sciences

For a reference copy of the document with all sections, see nature.com/documents/nr-reporting-summary-flat.pdf

Life sciences study design

All studies must disclose on these points even when the disclosure is negative.

Sample size	No sample size calculations was performed. This was a technology development study and human data generated are shown as a proof-of-concept of the the applicability of the pipeline.
Data exclusions	No data were excluded for this analysis.
Replication	All samples obtained from the mouse and human experiments were processed and run sequentially. All raw protein corona data were compiled and subjected to a harmonized analysis, aligning with recent recommendations in nanoparticle corona analysis. All attempts at replication were successful.
Randomization	All animals were randomized into either GBM or control group by card shuffle method. For human study randomization was not relevant as either patient or healthy control samples were recruited/selected for the purpose of the study.
Blinding	All animals were assigned animal number by someone who was not directly involved in this study. All corona/mass spec samples was allocated a sample number during sample processing rather than sample description.

Reporting for specific materials, systems and methods

We require information from authors about some types of materials, experimental systems and methods used in many studies. Here, indicate whether each material, system or method listed is relevant to your study. If you are not sure if a list item applies to your research, read the appropriate section before selecting a response.

Materials & experimental systems

n/a	Involved in the study	
x		Antibodies
	x	Eukaryotic cell lines
x		Palaeontology and archaeology
	x	Animals and other organisms
	x	Clinical data
x		Dual use research of concern
x		Plants

Methods

n/a	Involved in the study	
x		ChIP-seq
x		Flow cytometry
x		MRI-based neuroimaging

Eukaryotic cell lines

Policy information about cell lines and Sex and Gender in Research

Cell line source(s)	The murine GL261 cell line was obtained from the Leibniz Institute DSMZ, Germany.
Authentication	*Describe the authentication procedures for each cell line used OR declare that none of the cell lines used were authenticated.*
Mycoplasma contamination	Cell line was tested negative for mycoplasma contamination.
Commonly misidentified lines (See ICLAC register)	No commonly misidentified cell line used.

Animals and other research organisms

Policy information about studies involving animals; ARRIVE guidelines recommended for reporting animal research, and Sex and Gender in Research

Laboratory animals	Female C57BL/6J mice aged 12 weeks
Wild animals	The study did not involve wild animals
Reporting on sex	Findings apply only to female mice
Field-collected samples	Study did not involve field-collected samples
Ethics oversight	All procedures were performed in compliance with the United Kingdom Animals (Scientific Procedures) Act 1986 and local ethical approval under the Home Office project license P089E2E0A.

Note that full information on the approval of the study protocol must also be provided in the manuscript.

Clinical data

Policy information about clinical studies
All manuscripts should comply with the ICMJEguidelines for publication of clinical research and a completedCONSORT checklist must be included with all submissions.

Clinical trial registration	*Provide the trial registration number from ClinicalTrials.gov or an equivalent agency.*
Study protocol	*Note where the full trial protocol can be accessed OR if not available, explain why.*
Data collection	*Describe the settings and locales of data collection, noting the time periods of recruitment and data collection.*
Outcomes	*Describe how you pre-defined primary and secondary outcome measures and how you assessed these measures.*

Plants

Seed stocks

Report on the source of all seed stocks or other plant material used. If applicable, state the seed stock centre and catalogue number. If plant specimens were collected from the field, describe the collection location, date and sampling procedures.

Novel plant genotypes

Describe the methods by which all novel plant genotypes were produced. This includes those generated by transgenic approaches, gene editing, chemical/radiation-based mutagenesis and hybridization. For transgenic lines, describe the transformation method, the number of independent lines analyzed and the generation upon which experiments were performed. For gene-edited lines, describe the editor used, the endogenous sequence targeted for editing, the targeting guide RNA sequence (if applicable) and how the editor was applied.

Authentication

Describe any authentication procedures for each seed stock used or novel genotype generated. Describe any experiments used to assess the effect of a mutation and, where applicable, how potential secondary effects (e.g. second site T-DNA insertions, mosiacism, off-target gene editing) were examined.
